# A Systematic Review of the Use of Surgical Checklists in Transurethral Resection of Bladder Tumour

**DOI:** 10.3390/cancers16213626

**Published:** 2024-10-27

**Authors:** Abram Botros, Paul M. Rival, Ian D. Davis, Shomik Sengupta

**Affiliations:** 1 Faculty of Medicine, Nursing and Health Sciences, Eastern Health Clinical School, Monash University, Box Hill, VIC 3128, Australia; 2 Department of Urology, Eastern Health, Box Hill, VIC 3128, Australia; 3 Department of Oncology, Eastern Health, Box Hill, VIC 3128, Australia

**Keywords:** bladder cancer, transurethral resection of bladder tumour, systematic review, checklist

## Abstract

Context: Surgical checklists have previously been shown to improve surgical quality and patient outcomes. However, their use in transurethral resection of bladder tumour (TURBT), one of the most commonly performed urological procedures, has yet to be explored in depth. Objective: To evaluate the effect of surgical checklist implementation in TURBT on documentation quality, specimen quality, and oncological outcomes according to the existing literature. We then hope to develop an optimised TURBT checklist by identifying the most pertinent parameters for inclusion. Evidence acquisition: A literature search using PubMed was performed to identify literature pertaining to the use of surgical checklists in the context of TURBT. A systematic review was then performed on the 41 identified studies, of which six were included in the final analysis. Evidence synthesis: We explored three primary outcomes that arose from the literature, namely: (1) comprehensiveness of documentation; (2) resection quality; and (3) recurrence rates and recurrence-free survival (RFS). We found agreement in the literature that surgical checklist implementation does lead to an overall improvement in documentation. The effect of surgical checklists on resection quality and recurrence rates, however, was mixed in the literature, with some studies showing statistically significant improvements and others showing no significant change. Conclusions: There are multiple benefits to surgical checklist implementation in TURBT procedures. We propose an optimised 14-item surgical checklist that should be implemented in every TURBT report to ensure sufficient information documentation for risk stratification and post-operative management.

## Simple Summary

Transurethral resection of bladder tumour (TURBT) is a ubiquitous and essential procedure in the diagnosis and treatment of bladder cancer. Our systematic review of the use of standardised surgical checklists in this procedure has shown an overall improvement in oncological outcomes for bladder cancer patients. However, we found that the existing checklists were lacking in some areas. Based on our findings, we propose an optimised 14-item checklist for implementation in every high-quality TURBT.

## 1. Introduction

Bladder cancer (BC) represents a significant global health concern, with a rising incidence and prevalence over recent decades. According to the World Health Organisation (WHO), bladder cancer is the tenth most common cancer globally, accounting for an estimated 573,278 new cases and 212,536 deaths in 2020 [1]. Over 3500 new cases of BC were diagnosed in 2020 in Australia alone, accounting for approximately 2% of all cancer diagnoses in the country [2]. The management of BC demands a multimodal approach, which can comprise surgical intervention, chemotherapy or immunotherapy administration, cystoscopic surveillance, and diagnostic testing [3,4]. However, the journey for every BC patient will almost invariably begin with an index transurethral resection of bladder tumour (TURBT) procedure.

TURBT is a complex urological procedure crucial for the diagnosis, staging, and initial treatment of BC, whereby bladder tumours are endoscopically resected for oncological clearance and histopathological grading and staging [5,6]. Despite being a critical step in the management of BC, it is a procedure that also carries inherent risks, with significant complications such as bleeding, bladder perforation, and incomplete resection [7]. To mitigate these risks, enhance patient safety, and improve the quality of resection, the integration of surgical checklists has emerged in the literature as a potential strategy.

Surgical checklists represent structured tools designed to standardise and streamline procedural tasks, encouraging comprehensiveness and adherence to best practices and guideline recommendations [8]. Inspired by the aviation industry and popularised following the WHO Surgical Safety Checklist, checklists have repeatedly demonstrated their effectiveness in reducing surgical complications and improving patient outcomes across various specialties [9]. In the context of TURBT, surgical checklists may lead to multiple benefits, including improvements in resection quality, reduction of complications, procedural standardisation, and improved documentation [10].

Thus, this study aims to review the literature on the use of surgical checklists in TURBT to establish their impact and to develop a validated TURBT surgical checklist that can be implemented as a standard of care.

## 2. Evidence Acquisition

A comprehensive search of the PubMed database was performed using various combinations of the terms ‘TURBT’, ‘NMIBC’, and ‘Surgical checklist’ and their variant spellings and abbreviations (see Appendix A). Only studies from January 2000 to the time of review in August 2023 were searched, resulting in 41 papers. Titles and abstracts were then screened by two independent reviewers, where 22 were deemed irrelevant to the subject matter. Only studies that involved surgical checklist implementation in the context of TURBT and written in English were included. This left the remaining 19 studies for full-text review, of which six remained in the final analysis. The primary outcome was the effect of checklist implementation on the comprehensiveness of documentation, as defined by the change in recording rates of individual checklist items. The secondary outcomes were effects on rates of detrusor muscle inclusion in the resection specimen, and cancer recurrence—defined as an endoscopically or histologically confirmed tumour.

## 3. Evidence Synthesis

### 3.1. Checklist Inclusions

In total, twenty-one distinct checklist items were identified in the literature, with Anderson et al. [11] proposing three additional optional items that were not included in their final analysis. Table 1 shows the comprehensive list of checklist parameters in each of the six identified studies. There was a median of 9 (range: 8–12) items per finalised checklist. Four items were common across all identified checklists—tumour number, size, appearance, and completeness of resection. The next most common items were examination under anaesthesia (EUA) and visualisation of detrusor muscle (DM), which appeared in five of the six checklists.

Interestingly, none of the identified checklists included an item pertaining to satisfactory haemostasis, despite bleeding being a significant risk of this procedure [7]. Furthermore, some items of information necessary for post-operative non-muscle invasive bladder cancer (NMIBC) risk stratification were absent in some checklists. For example, tumour history was only present in three (50%) of the six checklists, and suspicion of CIS in four (66.7%) checklists.

### 3.2. Comprehensiveness of Documentation

Three studies specifically referenced the impact of surgical checklist implementation on documentation quality. According to Guerero et al., checklist implementation improved the proportion of operation notes deemed to be comprehensive according to European Association of Urology (EAU) standards from 1% to 22% [14]. Anderson et al. also found that surgical checklist implementation increased the average number of item inclusions per operative report from 4.8 to 8 out of 10 items (*p* < 0.001) [11]. The surgical checklist group also had a significantly larger proportion of reports that included all ten items—27% compared to 0.5% in the non-checklist group (*p* < 0.0001) [11]. Kikuchi et al. indicated that in their surgical checklist group, all nine critical parameters were recorded in 98% of procedures [15]. However, no reference was made to the non-checklist group’s completion rate for comparison.

Anderson et al. [11] and Guerero et al. [14] provide an in-depth breakdown of the documentation rate of each separate item pre- and post-checklist implementation. Notably, surgical checklists were associated with a statistically significant improvement in the recording rates of every item in Anderson et al.’s 10-item checklist (*p* < 0.0001 for each). Guerero et al. showed similar results, with 7 of 9 items showing a statistically significant rise in recording rates with checklist implementation. Of the two items that did not show a statistically significant improvement, tumour number had a recording rate of 100% in both the pre- and post-surgical checklist implementation groups, accounting for this result. Completeness of resection was the other statistically insignificant parameter due to similar recording rates pre- and post-checklist implementation (68% and 59%, respectively). These results can be found in Table 2 below.

Therefore, surgical checklist implementation leads to more parameter inclusions in operative reports and improved overall comprehensiveness of documentation.

### 3.3. Detrusor Sampling

One of the most widely accepted quality indicators in TURBT is the inclusion of DM in the resection specimen [17]. Muscle inclusion is considered a surrogate indicator of procedural quality as its absence in the resection specimen suggests an inadequately deep resection, which can lead to under-staging of disease and an increase in the risk of residual disease [18]. In fact, most guidelines mandate muscle sampling except in the case of small, solitary, Ta low-grade tumours [18]. In cases where muscle inclusion is not achieved, re-resection is often recommended [19].

The statistical significance of surgical checklist use on DM inclusion was mixed in the literature. Taoka et al. found that checklist use significantly increased the rate of muscle inclusion from 69.6% to 92% (*p* < 0.01) [13]. Moreover, on multivariate analysis of 48 specimens that did not contain DM, they found that checklist usage alone was independently associated with significant improvement in DM inclusion among a host of other intraoperative factors (odds ratio 4.78, 95% CI 2.19–10.4, *p* < 0.01) [13]. Likewise, a prospective study by Del Giudice et al. found that checklist completeness was significantly associated with DM presence in the specimen (odds ratio 1.23, 95% CI 1.12–1.35, *p* = 0.001) [16]. Furthermore, documentation of each additional item in their 9-item checklist increased the probability of DM sampling by 18% (odds ratio 1.18, 95% CI 1.06–1.31, *p* = 0.002) [16].

Conversely, some investigators found no statistically significant difference in DM inclusion pre- and post-checklist implementation. Kikuchi et al. and Suarez-Ibarrola et al. found that detrusor muscle inclusion rates remained similar pre- and post-checklist implementation ([90.90% vs. 90.2%, *p* = 0.086] and [62% vs. 59%, *p* = 0.15], respectively) [12,15]. Anderson et al. also found that there was no significant change in DM inclusion with checklist implementation (65% vs. 64%, *p* = 0.8) [11]. Still, they did note that regardless of checklist usage, the inclusion of more checklist items in operative reports resulted in a modest increase in DM inclusion, with an increase from 5 to 8 items associated with a 2.9% increase in the probability of muscle inclusion [11].

The evidence, therefore, seems to suggest that checklist implementation either leads to an improvement or no change in the rates of DM inclusion in resection specimens. None of the identified studies showed a detriment in DM inclusion with surgical checklist implementation, however.

### 3.4. Rates of Recurrence and Recurrence-Free Survival

Suarez-Ibarrola et al. found that recurrence-free survival (RFS) at 3-year follow-up was significantly lower when a surgical checklist was not used (*p* = 0.008) [12]. Furthermore, checklist use was independently associated with a significantly lower recurrence rate on multivariable analysis (Hazard Ratio 0.57, 95% CI 0.35–0.92; *p* = 0.02) [12]. Similarly, Taoka et al. showed a significant reduction in recurrence rate from 49.60% to 19.20% (*p* < 0.01) with checklist implementation [13]. Additionally, multivariate analysis using the statistically significant variables from the univariate analysis showed that lack of surgical checklist usage was the only factor independently associated with intravesical recurrence (hazard ratio 1.92, 95% CI 1.14–3.23, *p* = 0.01). At 12- and 24-months post-treatment, RFS rates were 82.8% and 77.2% in the checklist group and 65.5% and 50.8% in the non-checklist group, respectively (cumulative RFS log-rank test *p* < 0.001). In Kikuchi et al.’s study, a slight improvement in the 24-month RFS rates was noted between the checklist and non-checklist groups (76.7% and 69.5%, respectively) [15]. However, this did not reach conventional significance (*p* = 0.1059). Surgical checklist use, therefore, seems to lead to improved recurrence rates and RFS, with two of three studies showing statistically significant outcomes on this parameter.

## 4. Proposed Checklist

The ideal TURBT surgical operation report should serve to accomplish multiple objectives. Surgical documentation should contain sufficient descriptive information of the procedure and findings to enable accurate risk stratification, clinically stage the disease, assess the need for further post-operative resection, highlight actual or potential complications, and record whether post-operative intravesical chemotherapy has been administered [11,20,21]. In so doing, all the relevant information required to facilitate informed post-operative decision-making and management will be available. Additionally, the checklist should be comprehensive yet minimalistic enough to be user-friendly and conducive to workflow [22]. With these goals in mind, we set about establishing an optimised surgical checklist for use in TURBT procedures using the checklists from our literature review as a guide. We began by creating a list of subheadings under which items could be placed in order to address the themes identified above. These subheadings were (1) surgical indication, (2) tumour descriptors, (3) resection quality, (4) complications and (5) chemotherapy administration.

One consideration that was absent in the checklists from our systematic review was that of risk stratification. NMIBC guidelines often recommend a tier-based risk stratification of low-, intermediate- and high-risk bladder cancer, which relate to corresponding algorithms of treatment and surveillance [20,23,24]. These risk stratification groupings rely on a combination of histopathological and intraoperative findings documented in surgical operation reports [25]. Therefore, we considered including these intraoperative findings important when curating our checklist. The EAU NMIBC guidelines were carefully examined to distil a list of the minimum necessary intraoperative parameters required to facilitate accurate NMIBC risk stratification. This list included (1) tumour history, (2) size, (3) number, and (4) suspicion of concurrent CIS [20]. Tumour history specifically refers to whether the tumour in question is a primary tumour or a recurrence, and if recurrent, whether the recurrence is within 12 months and whether it was previously treated with intravesical chemotherapy. These were all included in our checklist.

Concerning clinical staging, we deemed EUA findings and the appearance of muscle invasion sufficient items. Although two studies from our systematic review included pre-operative and post-operative EUA as separate items, we could not find an explanation for why this would be pertinent. As such, we deemed a single pre-operative EUA sufficient for clinical staging.

Significant complication risks associated with TURBT are bleeding and bladder perforation [7,26]. As such, it is important for clinicians to document the adequacy of haemostasis at the end of the case and any concerns for bladder perforation following resection. This is of particular importance in the case of tumours at the anterior bladder and ureteric orifices, which are more prone to perforation and may require subsequent surgical correction [5]. Items pertaining to haemostasis and concern for perforation were therefore included in our checklist.

Given that oncological clearance is one of the primary aims of TURBT, intraoperative documentation should refer to the completeness of the resection [27]. Furthermore, the surgical report is often the sole indication of a residual tumour burden requiring repeat resection. In the case where tumours are difficult or dangerous to re-resect, documentation of this fact may also be important in preventing further futile attempts and could expedite a more pertinent discussion about radical treatment or palliation. Items pertaining to the location of the tumour, including the use of a bladder diagram as recommended in guidelines [20], and the use of specific technologies to identify them, such as photodynamic diagnosis (PDD) and narrow band imaging (NBI), were added. Furthermore, items that address the degree of macroscopic clearance and muscle inclusion were added to describe the resection quality for oncologic clearance and histopathological grading and staging.

Finally, given the existing evidence supporting the administration of postoperative single-dose intravesical chemotherapy, we felt this should be another noteworthy inclusion item [28]. The ideal chemotherapy agent, treatment duration, and administration frequency have not yet been established, as no randomised comparisons of specific chemotherapy agents have been performed [29]. A US study published in 2017 showed poor rates of post-operative single instillation of mitomycin in 32,0648 TURBTs, with only 53.1% of hospitals complying in a total of 6.1% of cases [30]. This specific parameter may, therefore, also be used judiciously as a general marker of guideline adherence.

As such, we developed a final optimised checklist comprising 12 critical items necessary to meet the criteria of the ideal surgical checklist for TURBT. The final items included were (1) tumour history, (2) tumour number, (3) size of largest tumour, (4) tumour shape, (5) presence of additional CIS, (6) appearance of muscle invasion, (7) pre-op EUA, (8) macroscopic clearance, (9) muscle visualisation at resection base, (10) adequacy of haemostasis, (11) concern for bladder perforation and (12) post-op intravesical chemotherapy instillation. The complete checklist, along with suggested responses, can be found in Table 3.

## 5. Conclusions

In summary, we propose an optimised 12-item surgical checklist for transurethral resection of bladder tumour procedures. This checklist is supported by our systematic review findings that surgical checklist implementation leads to improved documentation, resection quality, and recurrence rates, with varying results. Our checklist additionally factors in the minimum information necessary for NMIBC risk stratification, which was not addressed in the studies we identified for this review.

## Figures and Tables

**Table 1:** Comparison of checklist item inclusions for each of the six studies identified. (CIS = Carcinoma in situ, EUA = Examination under anaesthesia, DM = detrusor muscle).

	Anderson et al., 2016 [11]	Suarez-Ibarrola et al., 2019 [12]	Taoka et al., 2021 [13]	Guerero et al., 2022 [14]	Kikuchi et al., 2022 [15]	Del Giudice et al., 2023 [16]	TOTAL *n* (%)
**Patient Factors**							
(1) Tumour history (e.g., primary vs. recurrent)	Yes	Yes	No	No	Yes	No	3 (50%)
**Tumour Descriptors**							
(2) Tumour number	Yes	Yes	Yes	Yes	Yes	Yes	6 (100%)
(3) Tumour size	Yes	Yes	Yes	Yes	Yes	Yes	6 (100%)
(4) Tumour appearance	Yes	Yes	Yes	Yes	Yes	Yes	6 (100%)
(5) Tumour location (+/− drawing)	Optional	Yes	Yes	No	No	No	2 (33.3%)
(6) Appearance of muscle invasion	No	No	No	No	No	Yes	1 (16.7%)
(7) Surrounding mucosal changes/CIS	Yes	No	Yes	No	Yes	Yes	4 (66.7%)
(8) Clinical tumour stage	Yes	No	No	No	Yes	No	2 (33.3%)
(9) EUA (total)	-	-	No	-	-	-	5 (83.3%)
- EUA (pre-op)	-	Yes	-	Yes	-	-	2 (33.3%)
- EUA (post-op)	-	Yes	-	Yes	-	-	2 (33.3%)
- EUA (not specified)	Yes	-	-	-	Yes	Yes	3 (50%)
**Resection Quality**							
(10) Completeness of resection	Yes	Yes	Yes	Yes	Yes	Yes	6 (100%)
(11) Visualisation of DM in resection base	Yes	No	Yes	Yes	Yes	Yes	5 (83.3%)
**Complications**							
(12) Adequacy of haemostasis	No	No	No	No	No	No	0 (0%)
(13) Examination for bladder perforation	Yes	No	No	Yes	Yes	Yes	4 (66.7%)
**Chemotherapy administration**							
(14) Post-op mitomycin instillation	No	No	No	Yes	No	No	1 (16.7%)
**Other/Miscellaneous**							
(15) Photograph of resection sites	Optional	No	Yes	No	No	No	1 (16.7%)
(16) Separate deep biopsy from resection bed	Optional	No	No	No	No	No	0 (0%)
(17) Biopsy of prostatic urethra	No	No	No	No	Yes	No	1 (16.7%)
(18) Random biopsy of normal mucosa	No	No	No	No	Yes	No	1 (16.7%)
(19) Decision of a resection procedure	No	No	Yes	No	No	No	1 (16.7%)
**Total checklist items**	10 items [3 optional]	8 items	9 items	9 items	12 items	9 items	Mean: 9.5 items Median: 9 items Mode: 9 items

**Table 2:** Summary of the effect of surgical checklist implementation on recording rates, by parameter, as shown in Anderson et al., 2016 [11] and Guerero et al., 2022 [14]. (SC = surgical checklist, CIS = carcinoma in situ, EUA = examination under anaesthesia, DM = detrusor muscle).

Parameter	Anderson et al., 2016 [11]			Guerero et al., 2022 [14]		
	No SC *n* = 428 (%)	SC *n* = 325 (%)	*p* Value	No SC *n* = 78 (%)	SC *n* = 37 (%)	*p* Value
Primary vs. recurrent tumour	192 (45%)	257 (79%)	<0.0001	-	-	-
Tumour number	332 (78%)	303 (93%)	<0.0001	78 (100%)	37 (100%)	1
Tumour size	259 (61%)	286 (88%)	<0.0001	36 (46%)	33 (89%)	<0.05
Tumour appearance	292 (68%)	298 (92%)	<0.0001	46 (59%)	33 (89%)	0.001
Surrounding mucosal changes/CIS	160 (37%)	259 (80%)	<0.0001	-	-	-
Clinical tumour stage	77 (18%)	250 (77%)	<0.0001	-	-	-
EUA (pre-op)	194 (45%)	226 (70%)	<0.0001	37 (47%)	33 (89%)	<0.05
EUA (post-op)	-	-	-	11 (14%)	33 (89%)	<0.05
Completeness of resection	270 (63%)	268 (82%)	<0.0001	46 (59%)	25 (68%)	0.42
Visualisation of DM in resection base	126 (29%)	222 (68%)	<0.0001	28 (36%)	33 (89%)	<0.05
Examination for bladder perforation	171 (40%)	237 (73%)	<0.0001	17 (22%)	25 (68%)	0.001
Post-op mitomycin instillation	-	-	-	16 (21%)	20 (46%)	<0.05

**Table 3:** Our proposed checklist, derived from checklists in our literature review and EAU NMIBC guidelines on risk stratification. (N/A = parameter not applicable due to no visible tumour, UO = ureteric orifice, CIS = carcinoma in situ, EUA = examination under anaesthesia, DM = detrusor muscle).

Proposed TURBT Checklist	
Parameters	Responses
**Surgical indication**	
(1) Tumour history	[1]. Primary tumour [2]. Recurrent tumour [3]. Repeat TURBT [4]. Post intravesical therapy [5]. Other (specify)
a. If recurrent, recurrence within 12 months?	Yes|No
b. If recurrent, previous intravesical therapy?	Yes|No
**Tumour visual descriptors**	
(2) Tumour number	0|1|2–5|>5|Diffuse
(3) Size of largest tumour (cm)	<3 cm|≥3 cm|N/A
(4) Tumour shape	Sessile|Nodular|Papillary|Flat|N/A
(5) Tumour location	|Trigone|Right UO|Left UO|Right wall|Left wall|Anterior wall|Posterior wall|Dome|Bladder neck|Urethra|Other (specify)| OR [Free text description] OR [Bladder diagram (e.g., as per Babjuk et al., 2017 [20])]
(6) Presence of additional CIS	Suspicious|Not suspicious
(7) Appears muscle-invasive	Yes|No|N/A
(8) EUA	[1]. Palpable bladder tumour [2]. Palpable bladder tumour with extravesical extension [3]. Abnormal prostate (specify) [4]. No palpable tumour [5]. Not performed [6]. Other finding (specify)
**Resection quality**	
(9) Tumour visualisation technology	White light|Photodynamic diagnosis|Narrow band imaging|Other (specify)
(10) Macroscopically clear	Yes|No|N/A
(11) Detrusor muscle seen at resection base	Yes|No
**Complications**	
(12) Adequate haemostasis achieved	Yes|No
(13) Concern for bladder perforation	Yes|No|Not examined
**Chemotherapy administration**	
(14) Post-op chemotherapy instillation	Administered (specify agent)|Not administered

## Data Availability

The original contributions presented in the study are included in the article. Further inquiries can be directed to the corresponding authors.

## Appendix A. PubMed Search Terms

(“turbt” or “turb” or “transurethral resection of bladder tumor” or “trans-urethral resection of bladder tumor” or “transurethral resection of bladder tumour” or “trans-urethral resection of bladder tumour” or “bladder biopsy” or “bladder cancer” or “nmibc” or “non-muscle invasive bladder cancer” or “non muscle invasive bladder cancer”) and (“checklist” or “template” or “check-list” or “surgical checklist” or “standardised operation report” or “standardised operation note” or “standardized operation report” or “standardized operation note”).
